# Supplementary material for: Identification of photoperiod-induced specific miRNAs in the adrenal glands of Sunite sheep (Ovis aries)
Source: Front Vet Sci. 2022 Jul 22;9:888207. doi: 10.3389/fvets.2022.888207 (PMC9354845; doi:10.3389/fvets.2022.888207)
Supplement: Supplementary file 1 [file Data_Sheet_1.ZIP › Supplementary file/miRDeep2 file.zip!/miRDeep2 file/Know_Structure/SPLP42A1/oar-mir-370.pdf]

miRBase precursor : oar-mir-370
Total read count : 11981
oar-miR-370-5p read count : 42
oar-miR-370-3p read count : 11922
remaining reads : 17

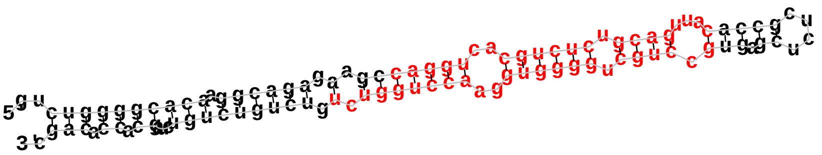

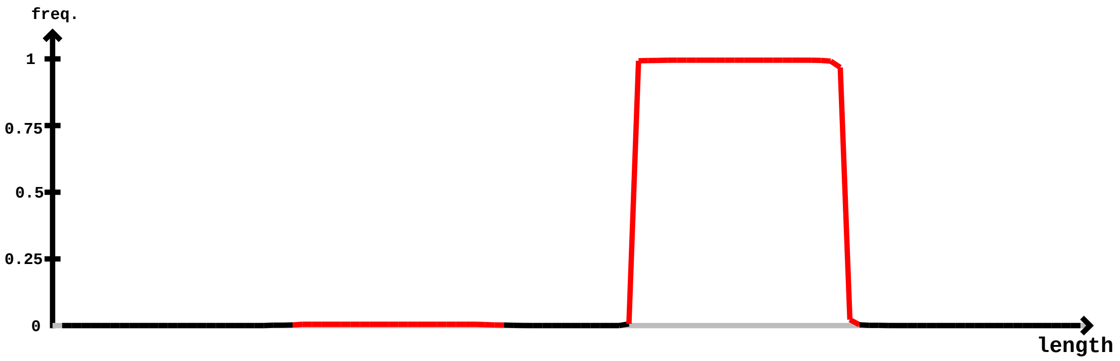


```
                                                                    oar-miR-370-3p
                                oar-miR-370-5p
5'- gucuggggcacaaggcagagaagccaggucacgucucugcaguuacaccgcucucgagugccugcuggggguggaaccuggucugucugucugucuagcaccacagc   -3'    exp
    ..((((((((((.((((((.(.(((((((..((((((.((((...(((((....)).))).)))).))))))..)))))))).).)))))))))...)).)).))).    reads    mm    sample
    .....................agccaggucacgucucugcagu................................................................    14       0     seq
    .....................agccaggucacgucucugcaguu...............................................................    3        0     seq
    .......................ccaggucacgucucugcaguu...............................................................    9        0     seq
    .......................ccaggucacgucucugcaguua..............................................................    1        0     seq
    .......................ccaggucacgucucugcaguuaca............................................................    1        0     seq
    ........................caggucacgucucugcagu................................................................    1        0     seq
    ........................caggucacgucucugcaguu...............................................................    5        0     seq
    ........................caggucacgucucugcaguua..............................................................    3        0     seq
    ........................caggucacgucucugcaguuac.............................................................    13       0     seq
    ........................caggucacgucucugcaguuaca............................................................    7        0     seq
    ........................caggucacgucucugcaguuacac...........................................................    2        0     seq
    ..........................................................ugccugcuggggguggaaccug...........................    1        0     seq
    ..........................................................ugccugcuggggguggaaccugg..........................    7        0     seq
    ..........................................................ugccugcuggggguggaaccuggu.........................    67       0     seq
    ..........................................................ugccugcuggggguggaaccugguc........................    1        0     seq
    ...........................................................gccugcuggggguggaacc.............................    1        0     seq
    ...........................................................gccugcuggggguggaaccu............................    10       0     seq
    ...........................................................gccugcuggggguggaaccug...........................    30       0     seq
    ...........................................................gccugcuggggguggaaccugg..........................    276      0     seq
    ...........................................................gccugcuggggguggaaccuggu.........................    11258    0     seq
    ...........................................................gccugcuggggguggaaccugguc........................    222      0     seq
    ...........................................................gccugcuggggguggaaccuggucu.......................    22       0     seq
    ............................................................ccugcuggggguggaaccuggu.........................    5        0     seq
    ............................................................ccugcuggggguggaaccugguc........................    4        0     seq
    ............................................................ccugcuggggguggaaccuggucu.......................    2        0     seq
    .............................................................cugcuggggguggaaccuggu.........................    2        0     seq
    .............................................................cugcuggggguggaaccugguc........................    3        0     seq
    .............................................................cugcuggggguggaaccuggucu.......................    1        0     seq
    ..............................................................ugcuggggguggaaccuggucugu.....................    8        0     seq
    ..............................................................ugcuggggguggaaccuggucuguc....................    1        0     seq
    ...............................................................gcuggggguggaaccuggucugu.....................    1        0     seq
```
